# Harnessing Synthetic Lethal Interactions for Personalized Medicine

**DOI:** 10.3390/jpm12010098

**Published:** 2022-01-12

**Authors:** Grace S. Shieh

**Affiliations:** 1 Institute of Statistical Science, Academia Sinica, Taipei 115, Taiwan; gshieh@stat.sinica.edu.tw; 2 Bioinformatics Program, Taiwan International Graduate Program, Academia Sinica, Taipei 115, Taiwan; 3 Genome and Systems Biology Degree Program, Academia Sinica, National Taiwan University, Taipei 115, Taiwan; 4 Data Science Degree Program, Academia Sinica, National Taiwan University, Taipei 115, Taiwan

**Keywords:** biomarker, cancer, genetic interaction, precision medicine, synthetic lethal

## Abstract

Two genes are said to have synthetic lethal (SL) interactions if the simultaneous mutations in a cell lead to lethality, but each individual mutation does not. Targeting SL partners of mutated cancer genes can kill cancer cells but leave normal cells intact. The applicability of translating this concept into clinics has been demonstrated by three drugs that have been approved by the FDA to target PARP for tumors bearing mutations in BRCA1/2. This article reviews applications of the SL concept to translational cancer medicine over the past five years. Topics are (1) exploiting the SL concept for drug combinations to circumvent tumor resistance, (2) using synthetic lethality to identify prognostic and predictive biomarkers, (3) applying SL interactions to stratify patients for targeted and immunotherapy, and (4) discussions on challenges and future directions.

## 1. Introduction

**Figure 1:**
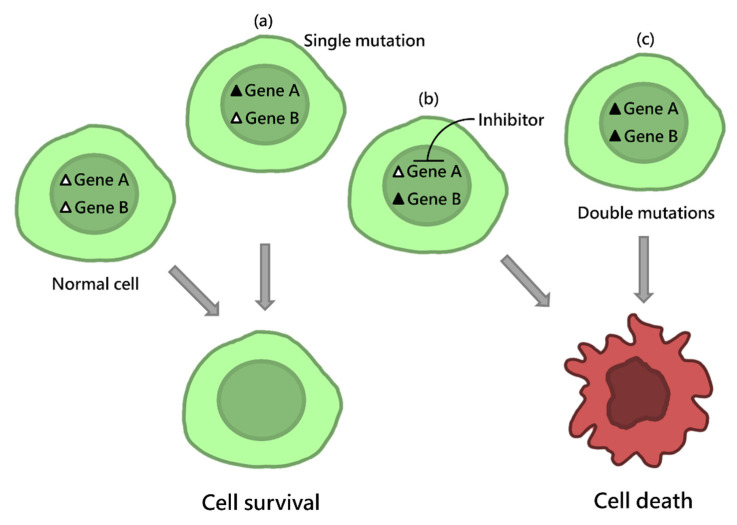
A graphic illustration of synthetic lethality. (**a**,**c**) Gene A and gene B are synthetic lethal when simultaneous mutation of gene A and B leads to cell death, but a single mutation of either does not. (**b**) The SL concept can be exploited to inhibit the SL partner (Gene A) of a mutant Gene B in a tumor cell.

Two genes are called synthetic lethal (SL, a type of genetic interaction [1]) when a simultaneous mutation of both genes leads to cell death, but a single mutation of either does not; see Figure 1 for an illustration. Although synthetic lethality was first observed in fruit flies by Calvin Bridges, the concept of synthetic lethality can be applied to exploit cancer-cell specific mutations for therapeutics as indicated in seminal papers [2,3]. Targeting SL partners of mutated cancer genes will selectively kill cancer cells but spare normal cells. Therefore, the synthetic lethality strategy offers a way to treat cancer cells with non-druggable mutant tumor suppressor genes (TSGs) and stability genes, e.g., *TP53* and *BRCA1*, by targeting their SL partners. The clinical relevance of synthetic lethality has been rapidly recognized. For example, pioneering studies of SL partners in *BRCA1* and *BRCA2*- deficient cancer cells identified *PARP1*. PARP inhibitors (PARPi) have become the first clinically approved drugs exploiting the synthetic lethality concept. The US FDA approved PARPi for ovarian cancer in 2014, breast cancer in 2018 and prostate cancer in 2020. Notwithstanding, it has taken more than 15 years since the concept of synthetic lethality was first indicated for cancer therapies [2] to develop these PARP inhibitors, which are used to treat breast cancer and high-grade serous ovarian cancer patients with homologous recombination deficiency (HRD), which includes mutation in *BRCA1/2*, *RAD51C/D*, or *PALB2,* hyper-methylation of the *BRCA1* promoter, or a series of yet to be defined causes [4]. It has been reported that PARP inhibitors are more effective for patients with *BRCA1*- and *BRCA2*- mutant ovarian cancer than for breast cancers [5], which shows that genetic context is crucial for functional genomic target screening. Advances in biotechnology such as CRISPR [6,7] have been expedited, which shall support the discovery of genetic contexts under which SL-based inhibitors work. Furthermore, there are many resources to mine novel SL interactions [8], e.g., Project DRIVE [9] and Project Achilles [10], which used a large set of human cell lines to uncover SL interactions, and multi-omics and clinical data of 33 cancer types at The Cancer Genome Atlas (TCGA). Additionally, a database named The Network Data Exchange (NDEx) [11] was built to organize the published SL interactions. As there are so many drug combinations, there are simply not sufficient patients to recruit for clinical trials, in addition to a huge amount of costs and time-consuming. Thus, adapting computational approaches, in particular machine learning-based approaches [11], will greatly benefit research on translating SL interactions to personalized medicine, as discussed later.

Here, we review applications of the SL concept to translational medicine in cancer over the past five years. In particular, we focus on the applications of synthetic lethality to personalized medicine. The scope of the article includes (1) exploiting the SL concept for drug combinations to circumvent tumor resistance, (2) using synthetic lethality to identify prognostic and predictive biomarkers, and (3) applying SL interactions to stratify patients for targeted and immunotherapy. We close with discussions on challenges and future directions.

## 2. Exploiting SL Interactions for Drug Combinations

Taking tumor heterogeneity into account, combinations of drugs will be more effective than single agent approaches [12]. Further, resistance may also be developed by a single targeted agent in a tumor, thus identification of effective drug combinations which target resistance pathways will also be crucial [13]. Experimentally validated SL pairs could lead to clinically relevant drug combinations provided that the genetic context of a tumor is well understood. This has been demonstrated by the success of PARP inhibitors and other SL combined drugs currently undergoing phase III clinical trials, e.g., olaparib combined with AR-pathway targeting in patients with metastatic castration-resistant prostate cancer (CRPCa) [14].

Jariyal and colleagues [15] reviewed several SL interactions which have been under clinical trial studies, in addition to olaparib and other PARP inhibitors. For example, Wee1 and TP53 is a known SL pair [16]. Hirai and colleagues showed that Wee1 inhibition (MK-1775) combined with DNA damage agents, such as gemcitabine, carboplatin, and cisplatin, led to apoptosis in p53-deficient cells [17]. This result provided a scientific basis for the phase II trial on MK-1775 in combination with carboplatin for patients with p53-mutant ovarian cancer [NCT01164995]. Moreover, *ATM* and *TP53* are also known SL. Durant and colleagues showed that ATM inhibitor (ATMi, AZD1390) combined with radiation therapy improved survival of preclinical brain tumor models [18], which led to a phase-1 clinical trial (NCT03423628). Thus, exploiting verified SL interactions to select patients with the associated mutations will optimize the patient outcomes and make clinical trials more efficient.

Currently, there are 342 clinical trial studies, registered at the US clinicaltrials.gov (accessed on 10 December 2021), for olaparib or olaparib-based drug combinations. In addition to breast cancer and ovarian cancer, there are also several clinical trials on PARP inhibitors for *BRCA1/2*- or *ATM*- mutant patients with prostate cancer [19] or cervical cancer [NCT04641728]. Recent studies of gene signatures for response to the PD-L1 inhibitor therapy (atezolizumab) in urothelial cancer (UC) [20,21,22], non-small cell lung cancer (NSCLC), and renal cell carcinoma (RCC) [23] revealed that the pathways most significantly associated with tumor mutation burden (TMB, a known factor correlated with response to immunotherapy checkpoint inhibitors), were cell cycle, DNA replication, and DNA damage response (DDR). DDR genes include *BRCA1/2*, *ATM*, *RAD51*, and others, and the former four genes are SL partners of PARP1. This provided a foundation for clinical trials on checkpoint inhibitors (CPIs) combined with PARPi in UC, NSCLC and RCC. Indeed, olaparib combined with pembrolizumab has been used for recurrent or metastatic cervical cancer patients in a phase II trial (NCT04641728). Several on-going clinical trials on combinations of olaparib and immunotherapies are listed in Table 1 (collected till 10 December 2021, from clinicaltrials.gov). Some studies include additional targeted therapies. We note that there is no phase III trial on combinations of olaparib and immunotherapy. However, there are phase III clinical trials on other PARP inhibitors, e.g., the JAVELIN Ovarian PARP 100 trial (NCT03642132), which failed due to no improvement of progression-free survival in patients. Additionally, another phase III clinical trial was withdrawn (NCT03806049).

Resistance also develops after patients are treated with PARP inhibitors, and resistance is important to the initiation of therapy. For example, olaparib prevents DNA repair. Thus, these tumor cells gain a growth advantage due to accumulated mutations, leading to clinical resistance to PARP inhibitors eventually [25]. Furthermore, most patients with advanced cancer eventually develop resistance to targeted therapies [26,27,28]. This acquired resistance may be treated by a secondary drug uncovered by synthetic rescue (SR) interactions [29]. Note that both primary and secondary resistance can be mediated by SR mechanisms. SR interaction refers to a functional interaction where a fitness reduction of cancer cells due to the inactivation of one gene, called a vulnerable gene, is compensated for by the altered expression of another, called a rescuer gene. Sahu and colleagues developed a computational approach (INCISOR), which successfully associated gene pairs with SR interactions. The inhibition of predicted rescuer genes sensitized resistant tumor cells to therapies, which was validated in vitro. Thus far, there is no clinical validation on SR interactions. The concept of SR-interactions has the potential for future basic research.

After effective drug combinations are discovered, these drug combinations could be used to identify patient populations that would respond. For example, prostate cancer patients with *BRCA1*- mutations could be selected for clinical trials and for treatments with olaparib and ATM inhibitor, provided that the drug combination is approved by the FDA. Note that the combination of olaparib and AZD0156 (an ATM inhibitor) is currently undergoing a phase-I clinical trial (CT02588105) for patients with advanced cancer.

## 3. SL Interactions to Uncover Prognostic and Predictive Biomarkers

In general, two types of biomarkers are investigated: prognostic and predictive [30]. A panel of prognostic biomarkers identified for cancers could enable the selection of patients best suited for intensive adjuvant therapies in clinics. Thus, prognostic biomarkers could help advance personalized medicine.

**Figure 2:**
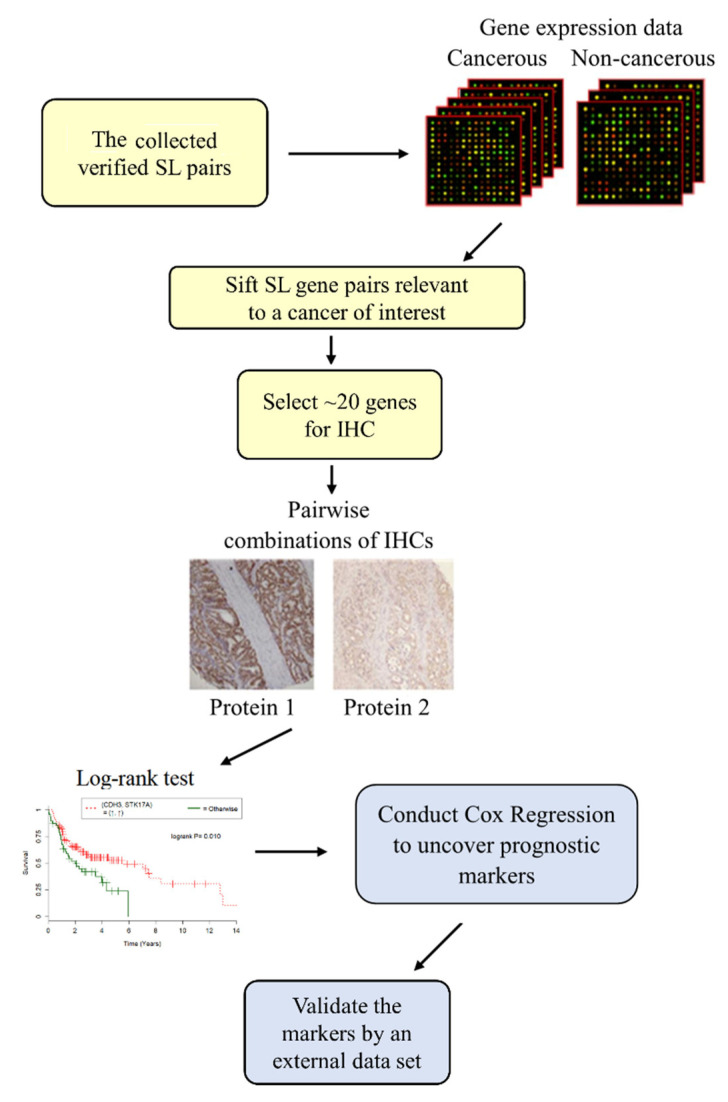
A graphical display of the approach in [31,32,33] to discover prognostic biomarkers. Gene expression of cancerous versus non-cancerous tissues was used to select SL gene pairs relevant to a cancer under study, from the collected SL pairs. This procedure resulted in ~20 genes for immunohistochemistry (IHC). Then combinations of IHC and overall survival of patients were analyzed by Cox regression to yield prognostic markers, which were further validated by at least one external data set such as TCGA.

Using published SL gene pairs, Shieh and colleagues developed a systematic approach to uncover IHC prognostic markers in colorectal cancer, lung adenocarcinoma and oral squamous cell carcinoma [31,32,33]. Specifically, they utilized a list of 643–742 SL pairs collected from the literature, gene expression data of the corresponding cancer under study, IHC expression, and clinical data on local cancer patients, to develop a computational approach to identify IHC prognostic biomarkers for the aforementioned three cancers. The authors first screened the collected SL pairs, most of which were validated by genome-wide RNAi screenings in various cancers [34,35], using microarray gene expression data of cancerous and non-cancerous tissues. They sorted the SL pairs by the fractions of the (up, up), (up, down), (down, up), and then (down, down) patterns, which were computed using patients’ gene expression of the associated cancers. About 20 genes with high fractions in the (up, up) pattern were selected for IHC staining. Next, they successfully identified single and combined IHC prognostic markers by correlating the single/paired IHC with overall survival of cancer patients via univariate Cox regression analysis [36]. The predicted prognostic markers were further verified by at least one external data set, e.g., TCGA lung adenocarcinoma for [32]. The approach revealed IHC marker pairs when neither single IHC was a marker. Furthermore, several of the identified prognostic markers with components involved in different pathways, e.g., the pair CK1e(C)-Rb1(N) was revealed to be a prognostic biomarker [33], but phosphorylation of CK1e is involved in the p53 pathway [37], which is different from the Rb pathway. As most of methods to uncover IHC markers to date have been mainly based on one or two proteins [38] or one pathway [39], their approach improved the current state-of-the-art for IHC markers. The flowchart of their approach is presented in Figure 2.

Srivas and colleagues [40] applied the conserved tumor suppressor genes (TSGs) from yeast to humans to identify TSGs interacting with the target of an FDA-approved drug. In particular, they identified ATM-irinotecan (inhibitor of TOP1 [41]). To date, both FOLFIRI (5-flourouacil plus irinotecan) and FOLFOX, which is a chemotherapy regimen made up of folinic acid, fluorouracil and oxaliplatin, have been indicated to treat metastatic colorectal cancer (mCRC) patients with an approximately 40% response rate. Nevertheless, there is no diagnostic test to guide selections from the aforementioned regimen for better response. Applying the SL combination *ATM* mutation and irinotecan, they found six out of 16 mCRC patients with *ATM* mutations, who were treated with irinotecan, had improved survival (44 months versus 29 months). Therefore, *ATM* could be a predictive biomarker to stratify mCRC patients for FOLFIRI [15].

## 4. Synthetic Lethality Applied to Stratify Patients for Targeted and Immunotherapy

### 4.1. Selection of Patients for Clinical Trials

Medical doctors determine whether new treatments are safe and more effective than current treatments through clinical trials. SL interactions can be applied to stratify patients for clinical trials of targets therapies as follows. In clinical trials of targeted drugs, if only a small proportion of patients have the required genetic context for response in a trial. Strong signals in individual patients will be diluted by patients without the necessary genetic context, and the trial may fail. In order to conduct clinical trials effectively, patients with proper genetic contexts should be selected. For example, de Bono and colleagues reported that only ~10% of patients enrolled showed mutations in the homologous recombination repair (HRR) biomarkers [42]. This lack of specificity poses a significant problem in clinical trials [19]. On the other hand, the first major biomarker study in prostate cancer (PCa) (the PROfound study) reported that 17.6% of the 4425 patients had mutations in at least one of the predefined 15 HRR genes, which included *BRCA1*, *BRCA2*, and *ATM* [42]. De Bono and colleagues revealed that Pca patients with *BRCA1*, *BRCA2,* or *ATM* mutations responded better to therapy and had increased progression-free survival and overall survival, whereas patients with long-tail HRR alterations such as *FANCL* or *RAD51C* did not have significant clinical benefits [43]. Analysis of DNA or/and RNA profiling of cancer patients and exploiting verified SL interactions will help prioritize candidates for clinical trials on SL-based drugs.

One example of how to select patients for a clinical trial using known SL pairs, e.g., *TP53-WEE1,* is the case of small cell lung cancer. As 100% of small cell lung cancer has the *TP53* mutation, it is expected that most small cell lung cancers have lost the G1 checkpoint and have a high probability of depending on Wee1 for proper DNA repair and cell cycle progression. Thus, we could select patients with p53-mutant small cell lung cancer for a clinical trial of Wee1 inhibitor, which is also an on-going phase II clinical trial (NCT026688907).

### 4.2. Synthetic Lethality Applied to Stratify Patients for Immunotherapy

Almost 90% of human cancer deaths are due to metastases. Immunotherapy is one of the most effective treatments for patients with certain metastatic cancer types to date. For instance, the PD-L1 inhibitor atezolizumab can treat certain patients with metastatic urothelial tumors [20,22]. Nevertheless, for some cancer types, e.g., HGSOC, CPIs did not work well [44]. Note that the objective response rate (ORR) for several cancers remain very low, for example ORR for urothelial cancer is only about 10% and less than 30% for non-small cell lung cancer. Thus, the identification of biomarkers to select patients for immunotherapy is important and very useful in clinical practice.

In addition to uncover biomarkers for checkpoint inhibitor immunotherapy, synthetic lethality can be exploited to direct immune cells specifically to tumor cells and destroy them as follows. The accumulation of genetic alterations in cancer cells, results in neoantigens. In theory, the immune system should generate T cell responses to recognize and kill nascent cancer cells. Nevertheless, tumor cells can escape immune pressure by evolving intrinsic genetic changes, for which [45] provided evidence. Zaretsk and colleagues found that loss of function mutations in *JAK1* could enhance immune evasion and confer anti-PD1 resistance in patients treated with a checkpoint inhibitor [45].

Recent studies also suggest that many oncogenes and TSGs may be involved in immune evasion. For example, *LKB1* was reported to be a putative tumor-intrinsic immune evasion gene [46]. Skoulidis and colleagues showed that *Lkb1/Stk11* loss promoted PD-1/PD-L1 inhibitor resistance, using *Kras*- mutant murine lung adenocarcinoma models. Furthermore, they found that patients with *LKB1* loss, correlating with reduced PD-L1 expression, did not respond to treatment with PD-1 inhibitors. This indicated that *LKB1* was a genuine suppressor of immune evasion. Other genetic alterations that correlated with immune evasion include *MYCN* amplification [47], *CASP8* loss of function [48], and *PTEN* loss of function [49]. *CASP8* loss of function was found to rescue cancer cells from T cell-killing by blocking the TNF pathway, while *PTEN* loss of function promoted resistance to T cell-mediated immunotherapy. Therefore, a functional evaluation of all known cancer genes may lead to the identification of drug targets to reverse immune evasion phenotype, and these targets can also serve as biomarkers to select patients for clinical trial of immunotherapy. For instance, the inhibition of over-expressed SL partner(s) of *LKB1, MYCN, CASP8,* and *PTEN* may reverse the immune evasion. Note that the identification of biomarkers through immune evasion targets has a great advantage, as biomarkers for immunotherapy have been difficult to find.

Discovery of immune evasion targets requires two procedures: (1) identification of a genetic context that gives rise to immune evasion, and (2) identification of drug targets that can reverse such immune evasion. As previously reviewed [5], SL-based CRISPR screening can be applied to tackle the first step. After an immune evasion genetic context has been uncovered, target screening can be performed in vitro with PD-L1 expression or other relevant immune readout, which may be the most efficient way to identify drug targets, as reported [5]. For example, identification of the immune evasion context for *JAK1* loss of function can be performed using cell lines harboring a *JAK1* loss of function mutation to measure PD-L1 expression after interferon stimulation. After the genetic context of immune evasion is revealed, SL-based target discovery approaches can be applied to discover the targets, which can reverse the immune evasion phenotype when knocked out. Advances in CRISPR technology [5] will enable the integration of cancer genetics (including the concept of synthetic lethality) and immune-oncology to elucidate the mechanism of immune evasion and make immunotherapy more clinically effective in the future.

## 5. Discussion and Future Directions

As mentioned in Section 1, there are too many drug combinations to recruit patients for clinical trials. Therefore, adapting computational approaches, followed by functional genomics screening to validate and confirm the SL interactions will be efficient. To achieve this aim, some useful computational algorithms and databases are available. Mining Synthetic Lethals (MiSL) [50] was developed to predict SL partners using multi-omics data, such as DNA mutation, copy number alteration, and gene expression, from 12 TCGA cancers. Sinha and colleagues exploited conserved TSGs from yeast to humans and used pancancer data to identify SL combinations [40]. The Network Data Exchange platform (NDEx) organizes published SL interactions [11] and is machine readable and searchable, so machine learning and database algorithms can be applied. As it encompasses a large volume of data, namely SL interactions in humans and other species and multi-omics data of ~33 cancer types, it will be efficient for predicting new SL interactions and the genetic context in which an SL-based drug will affect a particular cancer, through a machine learning approach. In addition to the big volume of omics data of various types of cancer, many validated genetic (including SL) interactions, protein interactions and prior knowledge are available, all of which can be used to train a machine learning algorithm. For instance, a deep learning algorithm was shown to predict drug combination effectively [51]. One can foresee that, once DNA sequencing and/or gene expression data of a patient’s tumor is profiled, and the data are fitted into a pre-trained machine learning algorithm, it will be possible to output the genetic context of the tumor and suggest an SL-based drug or a drug combination, in the near future. Then, a medical doctor can prescribe the suggested treatment to the patient accordingly. For example, olaparib combined with an immune CPI therapy can be prescribed to patients with *BRCA2*- mutant breast cancer.

Although Project DRIVE [9] and Project Achilles [10] have discovered SL interactions using a large set of human cell lines, the uncovered and fully verified drug targets remain limited. Project Score [52] has provided compelling evidence that there are still many drug targets for discovery, and they can be uncovered using a functional genomics approach. Synthetic lethality, ML-based computational algorithms, and the recent advances in biological science/technology, e.g., the powerful CRISPR-based functional screening, will enable the discovery of new SL interactions and new drug targets in cancer.

As SL-interactions work in a context-dependent fashion, the genetic context under which a targeted therapy or immunotherapy is added to the primary treatment (chemotherapy or targeted therapy) is critical to develop combined therapy. A future direction is to incorporate a computational approach to mine SL-based drug combinations, followed by validation using CRISPR, single cell techniques, and patient-derived organoids, which will enable the discovery of tumor heterogeneity underlying the primary drug resistance and secondary resistance driven by tumor cell evolution. This will eventually lead to combined drugs.

## Figures and Tables

**Table 1:** 16 clinical trials on combinations of olaparib and various immunotherapies; some studies include additional targeted therapies.

No.	Immunotherapy (and Targeted Therapies)	Investigated Cancer Types	Clinical Phase	Refs/Clinical Tal No.
1	Olaparib, AZD6738 or Durvalumab	TNBC ^1^	Phase II	NCT03740893
2	Olaparib and Pembrolizumab	Pancreatic Cancer	Phase II	NCT04548752
3	Olaparib, Durvalumab and Tremelimumab	Solid Cancers	Phase II	[24] NCT04169841
4	Olaparib and Pembrolizumab	Cervical Cancer	Phase II	NCT04483544
5	Olaparib and Pembrolizumab	TNBC	Phase II	NCT04683679
6	Olaparib and Pembrolizumab	Breast Cancer	Phase II	NCT03025035
7	Olaparib and Tremelimumab	Peritoneal Cancer, Fallopian Cancer and Ovarian Cancer	Phase II	NCT04034927
8	Olaparib, Nilotinib, Everolimus, Sorafenib, Lapatinib, Pazopanib, Durvalumab and Tremelimumab	Solid Neoplasms	Phase II	NCT02029001
9	Olaparib and Pembrolizumab	Pancreatic Cancer	Phase II	NCT05093231
10	Olaparib and Atezolizumab	Breast Cancer	Phase II	NCT02849496
11	Olaparib and Durvalumab	Prostate Cancer	Phase II	NCT04336943
12	Olaparib and Durvalumab	Bladder Cancer	Phase II	NCT04579133
13	Olaparib and Ramucirumab	Gastric Cancer Esophageal Cancer	Phase I/II	NCT03008278
14	Olaparib and Tremelimumab	Ovarian Cancer	Phase I/II	NCT02571725
15	Olaparib and Pembrolizumab	Melanoma	Phase II	NCT04633902
16	Olaparib and Bevacizumab, Cediranib and Cediranib Maleate	Glioblastoma	Phase II	NCT02974621

^1^ Note that TNBC denotes triple-negative breast cancer.

## Data Availability

Table 1 was queried from clinicaltrials.gov (accessed on 10 December 2021).
